# Poisoning for Burial Allowance from a Club

**Published:** 1848-09

**Authors:** 


*Poisoning for Burial Allowance from a Club.*—On Friday week last an inquest was concluded at Wickes, near Harwich, Essex, upon the body of a man, named Constable, a hawker, who lodged with his sister, a married woman, named May. He had been entered about a fortnight before his death by his sister as a member of a burial club, which paid £9 on the death of a member. He was then in excellent health. On the 8th ult., he returned home to tea, as usual, and was shortly after attacked with violent retching and burning pains in his throat and stomach. These symptoms continued till the following Sunday, when he died. The moment he was attacked, the sister called in several neighbours to see him. She assured them that it was almost certain he would die; but, strange to remark, no suspicion was excited that anything had been administered to him. On the declaration of the sister, that death had resulted from natural decline, the body was interred in the parish churchyard, she officiating as chief mourner. In the course of a few days she called on the Rev. G. Wilkins, the incumbent, for the purpose of obtaining a certificate that deceased was in good health a fortnight before his death, and that he was in his thirty-eighth year, (forty-eight was his real age.) This he declined giving, inquiring of her what she wanted it for? She replied that she had entered him in a burial club, at Harwich, a fortnight before his death, and that the society would not pay the money allowed for the interment of deceased unless they had a certificate of his good health at the time he was entered. He told her that the money did not belong to her. She said no one else was entitled to it, as she had done it all herself, and nobody else knew anything about it. These and other suspicious circumstances, particularly the number of children she had buried, coupled with the suspicious death of her former hurband, and the hasty and earnest solicitations she made in this instance to obtain the fees from the burial society, led to inquiry, and induced the coroner to direct the exhumation of the body. The stomach and contents were forwarded to Mr. Taylor, professor of Chemistry at Guy's Hospital, who discovered that a large quantity of arsenic had been administered to deceased. A great deal of circumstantial evidence having been adduced, showing an attempt on the part of Mrs. May to tamper with the principal witnesses, urging them not to disclose all they knew, the jury returned a verdict of "Wilful Murder" against the sister Mary May. She was committed to the county gaol for trial at the next assizes. The prisoner has been married twice, and had sixteen children, all of whom, with the exception of one, had died under suspicious circumstances. Their exhumation is expected to take place.—*Bell's Life in London.*

